# Baseline Lung Allograft Dysfunction After Bilateral Lung Transplantation Is Associated With an Increased Risk of Death: Results From a Multicenter Cohort Study

**DOI:** 10.1097/TXD.0000000000001669

**Published:** 2024-06-26

**Authors:** Michael B. Keller, Junfeng Sun, Muhtadi Alnababteh, Lucia Ponor, Pali D. Shah, Joby Mathew, Hyesik Kong, Ananth Charya, Helen Luikart, Shambhu Aryal, Steven D. Nathan, Jonathan B. Orens, Kiran K. Khush, Moon Kyoo Jang, Sean Agbor-Enoh

**Affiliations:** 1 Laborarory of Applied Precision Omics (APO), National Institutes of Health, Bethesda, MD.; 2 Laboratory of Transplantation Genomics, National Heart, Lung and Blood Institute (NHLBI), National Institutes of Health, Bethesda, MD.; 3 Genomic Research Alliance for Transplantation (GRAfT), Bethesda, MD.; 4 Division of Pulmonary and Critical Care Medicine, Johns Hopkins Hospital, Baltimore, MD.; 5 Critical Care Medicine Department, Clinical Center, National Institutes of Health, Bethesda, MD.; 6 Advanced Lung Disease Program and Lung Transplant Program, Inova Fairfax Hospital, Falls Church, VA.; 7 Division of Hospital Medicine, Johns Hopkins Bayview Medical Center, Baltimore, MD.; 8 Division of Pulmonary and Critical Care Medicine, University of Maryland Medical Center, Baltimore, MD.; 9 Genome Transplant Genomics (GTD), Stanford University School of Medicine, Palo Alto, CA.; 10 Division of Cardiovascular Medicine, Stanford University School of Medicine, Palo Alto, CA.; 11 Department of Pathology, Stanford University School of Medicine, Palo Alto, CA.

## Abstract

**Background.:**

A prior single-center, retrospective cohort study identified baseline lung allograft dysfunction (BLAD) as a risk factor for death in bilateral lung transplant recipients. In this multicenter prospective cohort study, we test the association of BLAD with death in bilateral lung transplant recipients, identify clinical risk factors for BLAD, and assess its association with allograft injury on the molecular level.

**Methods.:**

This multicenter, prospective cohort study included 173 bilateral lung transplant recipients that underwent serial pulmonary function testing and plasma collection for donor-derived cell-free DNA at prespecified time points. BLAD was defined as failure to achieve ≥80% predicted for both forced expiratory volume in 1 s and forced vital capacity after lung transplant, on 2 consecutive measurements at least 3 mo apart.

**Results.:**

BLAD was associated with increased risk of death (hazard ratio, 1.97; 95% confidence interval [CI], 1.05-3.69; *P* = 0.03) but not chronic lung allograft dysfunction alone (hazard ratio, 1.60; 95% CI, 0.87-2.95; *P* = 0.13). Recipient obesity (odds ratio, 1.69; 95% CI, 1.15-2.80; *P* = 0.04) and donor age (odds ratio, 1.03; 95% CI, 1.02-1.05; *P* = 0.004) increased the risk of developing BLAD. Patients with BLAD did not demonstrate higher log_10_(donor-derived cell-free DNA) levels compared with no BLAD (slope [SE]: –0.0095 [0.0007] versus –0.0109 [0.0007]; *P* = 0.15).

**Conclusions.:**

BLAD is associated with an increased risk of death following lung transplantation, representing an important posttransplant outcome with valuable prognostic significance; however, early allograft specific injury on the molecular level does not increase the risk of BLAD, supporting further mechanistic insight into disease pathophysiology.

Despite advances in donor selection, surgical techniques, and immunosuppression strategies, survival after lung transplantation remains the lowest of all solid organ transplants, with a median survival of 6.5 y.^1^ The primary goal of lung transplantation is to restore lung function with the purpose of improving quality of life and increasing survival. However, some lung transplant recipients do not attain normal lung function parameters following transplantation. This group of patients, categorized as baseline lung allograft dysfunction (BLAD), may represent a cohort of patients at increased risk of poor long-term outcomes, including chronic lung allograft dysfunction (CLAD) and death.^2^

A recent single-center retrospective cohort study demonstrated that BLAD was associated with an increased risk of death following lung transplantation.^2^ However, this association has not been evaluated in multicenter, prospective cohorts. Further, while early allograft injury in the form of primary graft dysfunction (PGD) has been associated with an increased risk of developing BLAD, the association of the degree of early allograft injury on the molecular level and the development of BLAD remains unknown.^3^ In this study, we aimed to test the hypothesis that BLAD is associated with an increased risk of death in lung transplant recipients. We further aim to test the hypothesis that the degree of early molecular allograft injury, as measured by donor-derived cell-free DNA (dd-cfDNA), is associated with an increased risk of BLAD.

## MATERIALS AND METHODS

We performed a multicenter, observational analysis that included bilateral lung transplant recipients >18 y old enrolled in 2 prospective cohort studies. The first study, Genome Transplant Dynamics (NCT01985412), enrolled lung transplant recipients at a single center (Stanford University Hospital) between December 1, 2010, and December 31, 2012, with follow up until May 1, 2019. The second study, Genome Research Alliance for Transplantation (NCT0243070), began enrollment in 2015 and is ongoing at 3 centers (Inova Fairfax Hospital, Johns Hopkins Hospital, and University of Maryland Medical Center). Both studies were designed similarly to evaluate the association of dd-cfDNA with various forms of allograft injury, including BLAD, CLAD, acute rejection, and infection. All patients underwent routine posttransplant monitoring with regularly scheduled clinic visits, lab draws, pulmonary function testing (PFT), and surveillance bronchoscopy with bronchoalveolar lavage and transbronchial biopsy. In addition, patients also underwent serially scheduled plasma sampling for dd-cfDNA on posttransplant days 1, 3, 7, 14, and 21 and at the time of all surveillance and for-cause bronchoscopies. We excluded patients who underwent single lung transplant, those who died <90 d posttransplantation, those with insufficient PFT data to adjudicate for BLAD, and those whose lung function did not reach a stable baseline (at least 1 follow up PFT >3 mo after their last PFT without a >20% increase in forced expiratory volume in 1 s [FEV1]). This study was approved by the Institutional Review Board at each participating center and the National Heart, Lung, and Blood Institute.

### Definition of Baseline Lung Allograft Dysfunction

BLAD was defined as failure to achieve ≥80% predicted for both FEV1 and forced vital capacity (FVC) after lung transplant, on 2 consecutive measurements at least 3 mo apart without a >20% increase in FEV1 in the 3 mo before their last recorded PFT values. The date of BLAD was considered the first date of the 2 measurements. The requirement for BLAD patients to not have a >20% increase in FEV1 on 2 consecutive PFT measurements >3 mo apart before their last recorded PFT was made to more confidently ensure that the patient’s lung function had plateaued and was not continuing to rise despite still being <80% predicted (as these patients continue to trend toward normal lung function and therefore do not have BLAD). Participants who attained ≥80% predicted FEV1 and FVC simultaneously on 2 consecutive measurements at least 3 mo apart were considered to have “normal” lung function, with the date of achieving normal lung function assigned as the first date of these 2 measurements. We further graded the severity of BLAD as follows based on prior studies: grade 1 (FEV1 ≥65% predicted), grade 2 (FEV1 50%–64% predicted), grade 3 (35%–49% predicted), and grade 4 (<35% predicted).^2,3^ Patients with BLAD grades 2–4 were classified as severe BLAD. Given that prior studies have demonstrated an association between obstructive spirometric function in the early posttransplant period with the development of CLAD, we further categorized BLAD participants as obstructive BLAD (BLAD-O) or restrictive BLAD (BLAD-R) based on having a ratio of FEV1:FVC ≥70% or <70%, respectively.^4^

### Outcomes

The primary outcome of this study was time to death. Secondary outcomes included time to CLAD and time to the composite endpoint of CLAD or death (whichever occurred first). CLAD was defined by International Society for Heart and Lung Transplantation criteria as a sustained decrease of ≥20% FEV1 from baseline value at least 3 mo posttransplant and taken at least 3 wk apart.^5^ PGD was defined according to International Society for Heart and Lung Transplantation criteria as the presence of bilateral pulmonary infiltrates consistent with noncardiogenic pulmonary edema 72 h posttransplant and graded according to the severity of hypoxemia as reflected by the ratio of Pao_2_ to fraction of inspired oxygen.^6^ Obesity was defined as a body mass index ≥30 kg/m^2^. Donor-recipient allograft size matching was defined by the ratio of the donor’s predicted total lung capacity (pTLC) to the recipient’s pTLC, with donor-recipient size mismatch defined as a donor pTLC: recipient pTLC ratio of <0.9 or >1.1.^7^

### Measurement of dd-cfDNA

Plasma dd-cfDNA was measured using an automated shotgun sequencing method as previously described.^8^ First, donor and recipient pretransplant genomic DNA was isolated and genotyped. Next, the recipient and donor genomes were compared with identify single-nucleotide polymorphisms (SNPs). After transplantation, recipient plasma cfDNA was isolated to generate a DNA library for paired-end shotgun sequencing. The cfDNA sequence reads were then interrogated for recipient and donor SNPs and dd-cfDNA was calculated as the percentage of donor SNPs to total (recipient plus donor) SNPs.

### Statistical Analysis

Categorical variables were summarized as counts and percentages, and compared using chi-square tests or Fisher exact tests. Continuous variables were summarized as mean (SD) or median (interquartile range [IQR]) and were compared using *t* tests or Wilcoxon rank-sum tests. Linear mixed models were used to analyze repeated measures of continuous variables. Standard residual diagnostics were used to check model assumptions. %dd-cfDNA was log-transformed unless nonparametric methods were used. Categorical outcomes were analyzed in multivariable logistic regression models to adjust for potential confounders. Covariates were prespecified based on the potential for influencing the outcome or evidence from prior studies demonstrating an increased risk of CLAD or death and included age, native lung disease, acute cellular rejection, PGD 3, and transplant center. Generalized estimating equations were used to account for repeated measures. Survival analysis was conducted using Cox proportional hazard models, and we confirmed no violation of the proportional hazard assumption. All analyses were performed using SAS, Version 9.4, and *P* values were 2-sided with a value of <0.05 indicating significance.

## RESULTS

**FIGURE 1.**
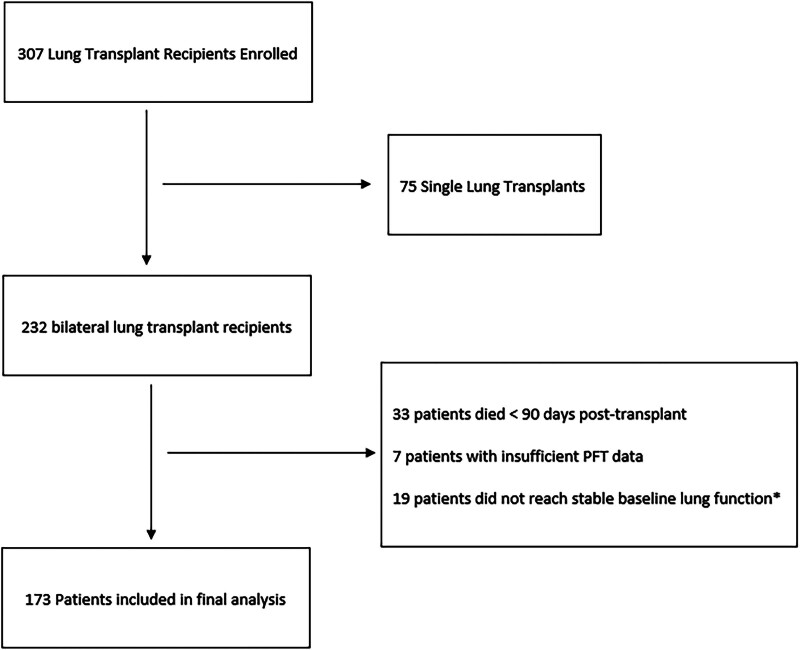
Flowchart of study design. PFT, pulmonary function testing.

Three hundred seven patients undergoing lung transplantation were enrolled during the study time frame. We excluded 75 patients who underwent single lung transplant, 33 patients who died before 90 d posttransplant, 7 patients who had insufficient PFT data to adjudicate for BLAD, and 19 patients whose lung function did not yet reach stable baseline, leaving 173 patients included in the final analysis (Figure 1). Median (IQR) duration of follow up was 29.0 mo (16.8–43.0 mo). The mean (SD) age at transplantation was 50.9 y (14.5 y), 45.1% of patients were female, and 82.1% were White (Table 1). The cumulative incidence of BLAD over the follow up period was 40.5%. The cumulative incidence of BLAD at 1 y was 30.6%.

**TABLE 1.** Demographic characteristics

	Total cohort (N = 173)	Normal function (N = 103)	BLAD (N = 70)	*P*
Recipient age (y), mean (SD)	50.9 (14.5)	53.1 (14.1)	47.5 (14.6)	0.01
Female recipient, N (%)	78 (45.1)	47 (45.6)	31 (44.3)	0.86
Recipient BMI (kg/ m^2^), mean (SD)	25.0 (4.7)	25.2 (4.1)	24.7 (5.5)	0.54
Obesity (BMI > 30 kg/ m^2^), N (%)	23 (13.3)	9 (8.7)	14 (20.0)	0.04
Recipient height (cm), mean (SD)	170 (9.09)	170 (8.01)	169 (10.6)	0.77
Native lung disease, N (%)				0.001
ILD	69 (39.9)	43 (41.8)	26 (37.1)	
COPD	36 (20.8)	29 (28.2)	7 (10.0)	
CF	40 (23.1)	23 (22.3)	17 (24.3)	
PAH	4 (2.3)	1 (1.0)	3 (4.3)	
Sarcoidosis	7 (4.1)	3 (2.9)	4 (5.7)	
Other	17 (9.8)	4 (3.9)	13 (18.6)	
Recipient race, N (%)				0.23
White	142 (82.1)	87 (84.5)	55 (78.6)	
Black	24 (13.9)	14 (13.6)	10 (14.3)	
Other	7 (4.1)	2 (1.9)	5 (7.1)	
Induction therapy, N (%)	85 (50.9)	52 (53.1)	33 (47.8)	0.51
PGD 3, N (%)	30 (17.8)	19 (19.0)	11 (15.9)	0.61
Perioperative ECMO (%)	8 (5.0%)	6 (6.4%)	2 (3.1%)	0.57
Index hospitalization length of stay, mean (SD)	22.8 (23.1)	18.4 (13.1)	31.3 (34.1)	0.086
Lung allocation score, mean (SD)	49.3 (17.1)	48.9 (17.6)	49.8 (16.7)	0.81
Donor age, mean (SD)	35.9 (13.9)	33.5 (12.9)	39.4 (14.6)	0.006
Donor smoking history, N (%)	10 (6.5)	4 (4.4)	6 (9.5)	0.32
Donor height (cm), mean (SD)	174 (9.80)	174 (10.5)	173 (8.68)	0.53
Recipient/donor size mismatch, N (%)	90 (52.0)	56 (54.4)	34 (48.6)	0.45
Undersized:	34 (19.7)	18 (17.5)	16 (22.3)	0.36
Normal match	83 (48.0)	47 (45.6)	36 (51.4)	
Oversized:	56 (32.4)	38 (36.9)	18 (25.7)	
Recipient predicted TLC (L), mean (SD)	6.04 (1.01)	6.07 (0.975)	6.00 (1.07)	0.67
Donor predicted TLC (L), mean (SD)	6.39 (1.02)	6.47 (0.995)	6.27 (1.05)	0.21
Acute cellular rejection (no. of patients), N (%)	62 (40.8%)	33 (37.1%)	29 (46.0%)	0.35
Early DSA (<30 d posttransplant, no. of patients), N (%)	33 (21.3%)	18 (19.6%)	15 (23.8%)	0.66

BLAD, baseline lung allograft dysfunction; BMI, body mass index; CF, cystic fibrosis; COPD, chronic obstructive pulmonary disease; DSA, donor-specific antibody; ECMO, extracorporeal membrane oxygenation; ILD, interstitial lung disease; PAH, pulmonary arterial hypertension; PGD, primary graft dysfunction; TLC, total lung capacity.

### Physiologic Characteristics of BLAD and BLAD Phenotypes

The mean (SD) baseline FEV1 and FVC of the cohort was 89.6% (21.3%) and 83.1% (17.9%) predicted, respectively. The median (IQR) time to baseline lung function of the cohort was 305 d (115–432 d). The mean baseline FEV1 and FVC for BLAD patients was 69.8% (14.5%) and 67.0% (14.3%) predicted, respectively, versus 103.1% (13.0%) and 93.9% (10.2%) predicted, respectively, for patients with normal pulmonary function. The median (IQR) time to baseline lung function for BLAD and normal function patients was 216 d (100–364 d) and 325 d (149–515 d), respectively (Table 2).

**TABLE 2.** Physiologic characteristics

	Total cohort (N = 173)	Normal function (N = 103)	BLAD (N = 70)	*P*
Time to baseline function (d), median (IQR)	305 (115–432)	325 (149–515)	216 (100–364)	0.01
FEV1 (L), mean (SD)	2.74 (0.83)	3.08 (0.73)	2.24 (0.71)	<0.0001
FEV1 (% predicted), mean (SD)	89.6 (21.3)	103.1 (13.0)	69.8 (14.5)	<0.0001
FVC (L), mean (SD)	3.31 (0.97)	3.68 (0.84)	2.77 (0.89)	<0.0001
FVC (% predicted), mean (SD)	83.1 (17.9)	93.9 (10.2)	67.0 (14.3)	<0.0001
FEV1/FVC ratio, mean (SD)	0.83 (0.09)	0.84 (0.07)	0.82 (0.11)	0.14

BLAD, baseline lung allograft dysfunction; FEV1, forced expiratory volume in 1 s; FVC, forced vital capacity; IQR, interquartile range.

In total, 15.7% of BLAD patients had BLAD-O physiology. The mean (SD) FEV1/FVC ratio in patients with BLAD-O was 0.63 (0.06). Median (IQR) time to baseline lung function was not significantly different in patients with BLAD-O compared with BLAD patients with BLAD-R physiology (244 d [113–359 d] versus 203 d [70–386 d]; *P* = 0.70). Patients with BLAD-O had lower FEV1 than BLAD-R patients (58.6 [15.2] versus 71.9 [13.5]; *P* = 0.005). BLAD-O had higher severity grades than BLAD-R (*P* = 0.02).

### Association of BLAD With Clinical Outcomes

**FIGURE 2.**
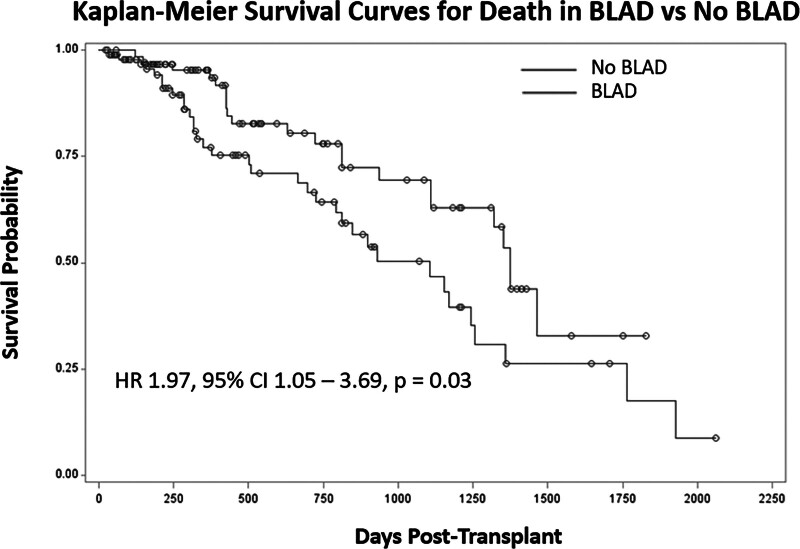
Kaplan-Meier survival curve comparing time to death in BLAD patients vs patients without BLAD. BLAD, baseline lung allograft dysfunction; CI, confidence interval; HR, hazard ratio.

Multivariable Cox regression analysis adjusting for age, native lung disease, acute cellular rejection, PGD 3, and transplant center demonstrated an increased risk of death in BLAD participants compared with participants with normal function (hazard ratio [HR], 1.97; 95% confidence interval [CI], 1.05-3.69; *P* = 0.03) (Figure 2). Participants with severe BLAD (grades 2–4) had impaired survival versus patients with normal lung function (HR, 3.07; 95% CI, 1.32-7.19; *P* = 0.01). The risk of death increased in a linear fashion with higher BLAD grades (HR, 1.70; 95% CI, 1.19-2.43; *P* = 0.004).

Patients with BLAD were at higher risk of the composite outcome of CLAD or death compared with patients with normal function (HR, 1.73; 95% CI, 1.02-2.95; *P* = 0.04). Patients with severe BLAD were also at higher risk of the composite outcome of CLAD or death compared with patients with normal function (HR, 2.83; 95% CI, 1.38-5.79; *P* = 0.005). Patients with BLAD were not at significantly higher risk of CLAD than patients without BLAD (HR, 1.60; 95% CI, 0.87-2.95; *P* = 0.13). There was no significant difference in the composite outcome of CLAD or death between BLAD-O and BLAD-R (HR, 0.89; 95% CI, 0.33-2.35; *P* = 0.81). There was no association with BLAD and development of acute rejection (*P* = 0.75).

### Risk Factors for BLAD

We performed multivariable logistic regression analysis to identify prespecified donor and recipient risk factors for the development of BLAD. Recipient obesity at the time of transplantation (odds ratio [OR], 1.69; 95% CI, 1.15-2.80; *P* = 0.04), native recipient lung disease designated as “other” (OR, 2.94; 95% CI, 1.06-8.19; *P* = 0.04), and donor age (OR, 1.03 per 1-y increase; 95% CI, 1.02-1.05; *P* = 0.004) were associated with an increased risk of developing BLAD (**Table S1, SDC**, http://links.lww.com/TXD/A670). Induction therapy (OR, 0.72; 95% CI, 0.54-0.95; *P* = 0.019) and chronic obstructive pulmonary disease (COPD) as the native lung disease (OR, 0.36; 95% CI, 0.16-0.79; *P* = 0.019) were associated with a decreased risk of developing BLAD. Neither grade 3 PGD at 48–72 h, donor smoking, donor-recipient size mismatch, or degree of donor-recipient HLA mismatch were associated with BLAD.

### Early Molecular Evidence of Allograft Injury and BLAD

For all patients, log_10_(dd-cfDNA) had a mean (SE) of 1.41 (0.03) on day 1 after lung transplant surgery and then decayed significantly during the first 90 d (slope [SE]: –0.0102 [0.0005]; *P* < 0.0001). Day 1 log_10_(dd-cfDNA) was 1.35 (0.04) for BLAD and 1.46 (0.03) for no BLAD (*P* = 0.054). Levels of log_10_(dd-cfDNA) at diagnosis of BLAD did not correlate with BLAD severity (*P* = 0.78; log_10_[dd-cfDNA] 0.23 [0.03] for grade 1, 0.27 [0.08] for grade 2, and 0.25 [0.12] for grades 3 and 4). Patients with BLAD did not demonstrate a significantly lower rate of reduction in log_10_(dd-cfDNA) levels over the first 90 d posttransplant compared with patient with normal pulmonary function (slope [SE]: –0.0095 [0.0007] versus –0.0109 [0.0007]; *P* = 0.15). Similarly, patients whose dd-cfDNA levels did not fall <1% in the first 90 d posttransplant did not show significantly higher risk of developing BLAD (41.5% versus 41.8%; *P* = 0.97). Levels of log_10_(dd-cfDNA) at the diagnosis of BLAD were not significantly higher for patients who experienced the composite outcome of CLAD or death versus not (0.22 [0.04] versus 0.28 [0.04]; *P* = 0.33). Levels of dd-cfDNA >1% at the diagnosis of BLAD were not significantly associated with an increased risk of the composite outcome of CLAD or death (46.2% versus 76.9%; *P* = 0.08).

## DISCUSSION

This multicenter, prospective cohort study demonstrates that bilateral lung transplant recipients with BLAD are at higher risk of death compared with those who have normal lung function. This study also identifies novel, independent risk factors for BLAD including obesity, donor age, and lack of induction therapy. Further, early allograft injury identified on the molecular level by dd-cfDNA did not increase the risk of BLAD. This study validates BLAD as an important prognostic factor in the risk stratification of lung transplant recipients and provides further mechanistic insight into the pathophysiological basis of disease.

Our findings are consistent with a prior single-center, retrospective cohort study by Liu et al^2^ that demonstrated an association of BLAD with an increased risk of death. Similarly, the authors of this study did not establish an association between BLAD and the development of CLAD. Notably, the study by Liu et al^2^ analyzed baseline dysfunction as a dynamic, time-varying covariate assessing the risk of death in patients with below normal lung function at regular intervals following lung transplant. Our definition of BLAD varied slightly, representing a binary variable assigned if a patient did not achieve normal lung function on 2 separate PFT values at least 3 mo apart and without a >20% increase in FEV1 before their last recorded PFT. Therefore, our definition attempts to more confidently exclude patients with lung function values below the normal threshold who may be demonstrating a normal posttransplant lung function trajectory and will ultimately not develop BLAD.

The association of BLAD with increased risk of death is not surprising, given that BLAD patients have lower baseline physiologic reserve. Episodes of acute rejection and infection are common after lung transplant, and patients with BLAD are likely more vulnerable to dying from these insults than patients with normal allograft function.^9-11^ Along these lines, our data do not show an increased risk of acute rejection in BLAD patients, yet it is plausible that these episodes of acute rejection are simply more consequential in patients with baseline graft dysfunction. The lack of association between BLAD and risk of CLAD may be explained by the potential for vulnerable BLAD patients to quickly progress to death as a result of an episode of acute lung allograft dysfunction rather than experience a progressive, stepwise decline in lung function sufficient for the diagnosis of CLAD (ie, these patients do not possess the reserve needed to tolerate such progressive declines in lung function before experiencing death).

Recipient obesity and donor age were found to be significant independent risk factors for BLAD in our multivariable model. Prior studies have consistently revealed obesity as a significant risk factor for poor posttransplant survival; however, reports of the effects of obesity on posttransplant lung function have been limited.^12-14^ The mechanisms underlying the association of obesity with BLAD remained undefined, but several possibilities exist. Impairment of lung function because of obesity can be attributed to mechanical or pro-inflammatory factors. Obesity results in reduced compliance of the respiratory system, primarily through a reduction in chest wall compliance.^15,16^ The effects of obesity on pulmonary function may depend on the distribution of fat deposition, with central adiposity (primarily in the thorax and abdomen) having a more significant effect on lung function than peripheral adiposity.^17-19^ Further, prior studies have demonstrated that obesity may induce pro-inflammatory effects on the lung mediated by the pro-inflammatory effects of leptin.^20,21^ Animal models have also shown that leptin may promote the progression of lung fibrosis after episodes of acute lung injury by augmenting the production of tumor growth factor beta signaling in lung fibroblasts and inhibiting peroxisome proliferator-activated receptor gamma,^22^ providing another potential link of obesity to BLAD.

The effects of donor age on posttransplant outcomes have been met with mixed results; however, few studies have evaluated the effects of donor age on posttransplant spirometry.^23,24^ Prior studies indicate that advanced donor age (≥70 y old) is associated with impaired posttransplant spirometric indices, with minimal effect on survival.^25,26^ Our cohort did not contain any recipients with a donor age of >65 y old. Although the relationship between donor age and BLAD is plausible, given that lung function tends to decline with age, future studies should evaluate the impact of specific age thresholds and donor-recipient age mismatch on the risk of BLAD.

Both COPD as an indication for transplantation and induction therapy were independently associated with a reduced risk of BLAD. Patients with COPD have larger thoracic cavity volumes (TCVs) at the time of transplant than patients with native restrictive lung disease.^27^ Although posttransplant chest wall remodeling tends to decrease TCV in patients with native obstructive lung disease, TCV at 1-y posttransplant remains higher than their native restrictive counterparts and is associated with higher FEV1.^27^ Prior studies investigating the effects of induction therapy on posttransplant survival have had inconsistent results; however, several studies suggest a survival benefit.^28-30^ Our data suggest this may, in part, be associated with a reduced risk of BLAD, but additional studies are required to confirm this association. Further, the pathophysiological mechanisms underlying the association of lack of induction therapy with BLAD remain unclear, especially given the lack of association between PGD 3, acute rejection, and molecular allograft injury on the development of BLAD. Additional studies are needed to investigate this association.

The lack of association between grade 3 PGD and risk of BLAD in our cohort conflicts with a prior study performed by Li et al.^3^ This may be because of methodologic differences secondary to the longer required follow up time of 3 mo without an associated increase in FEV1 to confirm BLAD. Prior studies in survivors of acute respiratory distress syndrome indicate that lung function tends to progressively rise over the year following diagnosis and reach normal spirometric values, suggesting that adequate follow up time may be necessary to ensure that patient lung function has plateaued.^31^ Perhaps more plausible, is that our study was underpowered to detect an association between PGD and BLAD, consistent with a prior study of a smaller cohort size also failing to demonstrate an association between PGD and BLAD.^2^ It is notable that patients with BLAD had longer index hospitalizations, although this did not reach statistical significance, suggesting that more frequent or severe posttransplant complications, or prolonged posttransplant recoveries, may contribute to the development of BLAD.

We expected, but did not find, an association between the degree of early allograft injury on the molecular level and the development of BLAD. This was surprising considering prior studies demonstrating an association with the degree of molecular allograft injury and posttransplant survival and CLAD.^32,33^ There are several possible explanations for these finding. Plasma dd-cfDNA originates directly from injury to the allograft itself; however, BLAD likely represents a highly heterogeneous process characterized by both parenchymal and/or extraparenchymal lung disease. Conditions such as neuromuscular weakness, pleural effusions, bronchial complications, or chest wall restriction may result in an impairment of pulmonary function with less parenchymal involvement of the allograft. This is supported by our findings of obesity as a risk factor for BLAD, assuming the purely mechanical effects of obesity are the underlying mechanism. Further, Darley et al^34^ demonstrated an increased incidence of pleural thickening or pleural effusion in patients with BLAD versus those without BLAD, which may result in a decline in lung function without necessarily increasing levels of dd-cfDNA. Although levels of dd-cfDNA tend to correlate with acute declines in lung function, it is also possible that the degree of allograft injury on the molecular level influences long-term outcomes in the absence of significant effects on baseline pulmonary function.^35^ Future studies evaluating the association of allograft injury on the molecular level and BLAD, while stratifying for the presence of extrapulmonary pathology, would provide further insight.

Although this study offers novel insight into the risk factors associated with the development of BLAD and validates its association with poor long-term outcomes, several limitations exist. Given the observational nature of the study, residual confounding may be present despite the use of Cox regression models incorporating established risk factors for CLAD and death. Measures of respiratory muscle strength, incidence of diaphragmatic paralysis or body mass index at the time of BLAD diagnosis were not captured in our cohort. Further, duration of mechanical ventilation in the early posttransplant setting, need for reintubation, and ICU length of stay were not captured in our study. A prior study by Mohanka et al^36^ demonstrated that these factors were associated with lower FVC in the first-year posttransplant. Because of limited sample size and lack of lung volume/radiographic data, we were unable to correlate specific phenotypes of BLAD-O and BLAD-R with more granular phenotypes of CLAD such as restrictive allograft syndrome, undefined, and mixed phenotypes. Although our study focused on the outcome of death and CLAD, future studies exploring the relationship of BLAD to other patient-oriented outcomes such as quality of life or hospital-free days would be useful.

In conclusion, BLAD is associated with an increased risk of death following lung transplantation. Recipient obesity, donor age, and lack of induction therapy are potential risk factors for the development of BLAD while early allograft injury on the molecular level is not. This study supports the use of BLAD as a relevant patient outcome in the posttransplant setting with important prognostic significance, warranting future studies aimed at identifying pathophysiological mechanisms, phenotypes, preventative strategies, and treatment.

## Supplementary Material


