# Supplementary material for: Baseline Lung Allograft Dysfunction After Bilateral Lung Transplantation Is Associated With an Increased Risk of Death: Results From a Multicenter Cohort Study
Source: Transplant Direct. 2024 Jun 26;10(7):e1669. doi: 10.1097/TXD.0000000000001669 (PMC11216668; doi:10.1097/TXD.0000000000001669)
Supplement: Supplementary file 1 [file txd-10-e1669-s001.pdf]

Supplementary Index

Baseline lung allograft dysfunction after bilateral lung transplantation is associated with an increased risk of death: Results from a multicenter cohort study

Michael B. Keller, Junfeng Sun, Muhtadi Alnababteh, Ileana L. Ponor, Pali D. Shah, Joby Mathew, Hyesik Kong, Ananth Charya, Helen Luikart, Shambhu Aryal, Steven D. Nathan, Jonathan B. Orens, Kiran K. Khush, Moon Kyoo Jang, Sean Agbor-Enoh

Table S1: Clinical Risk Factors for BLAD							
Analysis Of GEE Parameter Estimates							
**Parameter**		**Estimate**	**Standard Error**	**95% Confidence Limits**		**Z**	**Pr > |Z|**
**Intercept**		0.2736	0.5737	-0.8509	1.3981	0.48	0.6334
**PGD3**	1	-0.3994	0.5059	-1.3910	0.5922	-0.79	0.4299
**PGD3**	0	0.0000	0.0000	0.0000	0.0000	.	.
**Obese**	1	0.5225	0.2596	0.0137	1.0313	2.01	0.0442
**Obese**	0	0.0000	0.0000	0.0000	0.0000	.	.
**Native Lung Disease**	COPD	-1.0352	0.4092	-1.8372	-0.2332	-2.53	0.0114
**Native Lung Disease**	Cystic Fibrosis	-0.6179	0.8476	-2.2791	1.0433	-0.73	0.4660
**Native Lung Disease**	ILD	-0.2944	0.9868	-2.2286	1.6398	-0.30	0.7654
**Native Lung Disease**	Other*	1.0792	0.5227	0.0548	2.1036	2.06	0.0389
**Native Lung Disease**	PAH	0.4936	1.2991	-2.0526	3.0399	0.38	0.7040
**Native Lung Disease**	Sarcoidosis	0.0000	0.0000	0.0000	0.0000	.	.
**Donor-Recipient Size Mismatch**	1	-0.2378	0.4749	-1.1685	0.6929	-0.50	0.6165
**Donor-Recipient Size Mismatch**	0	0.0000	0.0000	0.0000	0.0000	.	.
**Donor Smoking Hx**	Yes	-0.0992	0.3528	-0.7907	0.5923	-0.28	0.7786
**Donor Smoking Hx**	No	0.0000	0.0000	0.0000	0.0000	.	.
**Age**		-0.0252	0.0167	-0.0579	0.0075	-1.51	0.1315
**Donor Age**		0.0335	0.0095	0.0149	0.0522	3.52	0.0004
**Female**	1	0.1861	0.3243	-0.4495	0.8217	0.57	0.5660
**Female**	0	0.0000	0.0000	0.0000	0.0000	.	.
**Induction**	1	-0.3343	0.1420	-0.6126	-0.0559	-2.35	0.0186
**Induction**	0	0.0000	0.0000	0.0000	0.0000	.	.
**HLA Mismatch_>4**	1	-0.1596	0.2546	-0.6586	0.3394	-0.63	0.5308
**HLA Mismatch_≤4**	0	0.0000	0.0000	0.0000	0.0000	.	.

***Other native lung disease includes alpha-1-antitrypsin deficiency, pulmonary veno-occlusive disease, non-CF bronchiectasis, alveolar proteinosis, and bronchiolitis obliterans syndrome.**
